# *Sarcocystis cruzi* (Hasselmann, 1923) Wenyon, 1926: redescription, molecular characterization and deposition of life cycle stages specimens in the Smithsonian Museum

**DOI:** 10.1017/S003118202300094X

**Published:** 2023-11

**Authors:** J. P. Dubey, Aditya Gupta, Larissa S. de Araujo, Oliver C. H. Kwok, Asis Khan, Benjamin M. Rosenthal

**Affiliations:** United States Department of Agriculture, Agricultural Research Service, Animal Parasitic Diseases Laboratory, Beltsville Agricultural Research Center, Beltsville, MD 20705-2350, USA

**Keywords:** bison, cattle, history, life cycle, molecular, *Sarcocystis cruzi*

## Abstract

Currently, 7 named *Sarcocystis* species infect cattle: *Sarcocystis hirsuta*, *S. cruzi*, *S. hominis*, *S. bovifelis*, *S. heydorni*, *S. bovini* and *S. rommeli*; other, unnamed species also infect cattle. Of these parasites of cattle, a complete life cycle description is known only for *S. cruzi*, the most pathogenic species in cattle. The life cycle of *S. cruzi* was completed experimentally in 1982, before related parasite species were structurally characterized, and before the advent of molecular diagnostics; to our knowledge, no archived frozen tissues from the cattle employed in the original descriptions remain for DNA characterization. Here, we isolated DNA from a paraffin-embedded kidney of a calf experimentally infected with *S. cruzi* in 1980; we then sequenced portions of 18S rRNA, 28S rRNA, COX1 and Acetyl CoA genes and verified that each shares 99–100% similarity to other available isolates attributed to *S. cruzi* from naturally infected cattle. We also reevaluated histological sections of tissues of calves experimentally infected with *S. cruzi* in the original description, exploiting improvements in photographic technology to render clearer morphological detail. Finally, we reviewed all available studies of the life cycle of *S. cruzi*, noting that *S. cruzi* was transmitted between bison (*Bison bison*) and cattle (*Bos taurus*) and that the strain of parasite derived from bison appeared more pathogenic than the cattle strain. Based on these newfound molecular, morphological and physiological data, we thereby redescribed *S. cruzi* and deposited reference material in the Smithsonian Museum for posterity.

## Graphical abstract

**Figure:**
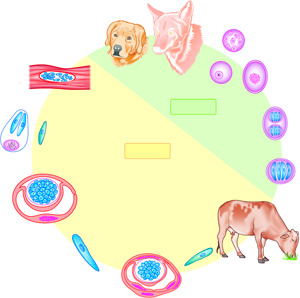

## Introduction

Cattle are routinely infected with parasites in the genus *Sarcocystis*. The taxonomy of *Sarcocystis* species in cattle remains debated; at least 7 named species occur: *S. cruzi*, *S. heydorni*, *S. bovini*, *S. hirsuta*, *S. rommeli*, *S. hominis* and *S. bovifelis*. Molecular evidence suggests the occurrence of additional species (reviewed in Dubey and Rosenthal, 2023). Among these species of bovine *Sarcocystis*, a complete description of the life cycle is known only for *S. cruzi*. The life cycle of *S. cruzi* was completed in 1982 (Dubey, 1982*a*) experimentally in cattle before the advent of molecular diagnostics (Dubey *et al*., 1989); to our knowledge, no archived frozen tissues from the cattle employed in the original descriptions remain for DNA characterization. Current DNA characterization is based on naturally infected cattle (Rosenthal *et al*., 2008; Gjerde, 2016; reviewed in Dubey and Rosenthal, 2023). For genotyping, several genes have been employed to characterize *S. cruzi* from naturally infected cattle including small subunit (SSU) ribosomal RNA (18S rRNA) (Holmdahl *et al*., 1993; Rosenthal *et al*., 2008; Jehle *et al*., 2009; Gjerde, 2016; Gjerde *et al*., 2016), large subunit ribosomal RNA (28S rRNA) (Gjerde, 2016); internal transcribed spacer of rDNA (ITS1 and ITS2) (Rosenthal *et al*., 2008; Gjerde, 2016) and mitochondrial genes, cytochrome c oxidase subunit (COX1) (Gjerde, 2013, 2016; Gjerde *et al*., 2016). Surveys based on these were summarized previously (Dubey *et al*., 2016; Dubey and Rosenthal, 2023). Additionally, Doi *et al*. (2023) reported acetyl-CoA synthetase gene marker in *S. cruzi* from the cardiac muscles of cattle from Saitama Prefecture, Japan.

Here, we reevaluated studies on the life cycle of *S. cruzi*, identified additional morphological details of the developmental stages, redescribe the parasite, provide the first molecular characterization from experimentally infected cattle and deposit life cycle stages in the Smithsonian Museum for future reference.

### History and background

Before the discovery of the obligatory 2-host life cycle of *Sarcocystis* species in 1972, only 1 species of *Sarcocystis* (*Sarcocystis fusiformis*) was recognized in cattle (Heydorn and Rommel, 1972; Rommel and Heydorn, 1972; Rommel *et al*., 1972). Then, researchers believed *S. fusiformis* parasitized both cattle (*Bos taurus*) and water buffalo (*Bubalus bubalis*). Currently, the name *S. fusiformis* is restricted to the macroscopic sarcocyst-forming species in water buffalo (*B. bubalis*); no species of *Sarcocystis* sharing its morphology has yet been identified in cattle (Dubey *et al*., 2014, 2015*b*).

Subsequent investigations in Germany identified 3 species of *Sarcocystis* infecting cattle: one transmitted exclusively by canids, one exclusively by cats and the third exclusively by humans. Based on these findings, Heydorn *et al*. (1975*b*) proposed new names for species of *Sarcocystis* in cattle; *S. bovicanis* for the species transmitted *via* dogs, *S. bovifelis* for the species transmitted by cats and *S. bovihominis* for the species transmitted by humans. An intense debate followed the proposal to rename these species. Scientists from Germany and their collaborators (reviewed in Dubey, 2022) sought a rational system, combining information from the intermediate and definitive host (e.g. *S*. *bovicanis*). They suggested new names for *Sarcocystis* species of livestock (reviewed in Dubey, 2022). However, Levine (1977) upheld the principle of nomenclatural priority. This principle seeks stability in scientific communication by retaining valid names for described species. Levine (1977) concluded that the previously used names for *Sarcocystis* species must be retained, even though the descriptions of species were incomplete; he assigned *S. cruzi* for *S. bovicanis*, *S. hirsuta* for *S. bovifelis* and *S. hominis* for *S. bovihominis*.

### History and biology of *S. cruzi*

Levine (1977) credited Hasselmann (1926) with naming *S. cruzi*. Levine was unaware of an earlier paper by Hasselmann who named it as *Miescheria cruzi* in a brief note in 1923 (reviewed in Dubey and Rosenthal, 2023). Neither of these papers by Hasselmann (1923, 1926) contains a morphological description of the parasite. Hasselmann found the parasite in all 55 hearts from Salvador (Bahia) and São Paulo, Brazil (Hasselmann, 1923). He called the parasite *cruzi* because it resembles *Trypanosoma cruzi* in histological sections. Hasselmann was unaware of *Sarcocystis* species in cattle. Wenyon (1926) transferred the genus *Miescheria* to the genus *Sarcocystis*, resulting in *Sarcocystis cruzi* (Hasselmann, 1923). Hasselmann, a pathologist, focused on host reactions rather than parasite morphology. In his 1926 paper, Hasselmann (1926) called the parasite *Miescheria crusi* but the name *cruzi* has precedence over *crusi* (Dubey and Rosenthal, 2023). *Sarcocystis cruzi* sarcocysts are thin walled (<1.0 *μ*m) and commonly parasitize the myocardium. Until 2014, all sarcocysts with thin walls were considered *S. cruzi*. In 2015, a new thin-walled species, *S. heydorni*, was recognized; importantly, its definitive host is human, not canid (Dubey *et al*., 2015*a*).

Most experiments involving the life cycle of *S. cruzi* were performed in Germany and USA; here we review these studies.

## Experimental transmission of bovine *Sarcocystis* to dogs in Germany

In the pioneering study by scientists in Germany, muscles from cattle oesophagi naturally infected with *Sarcocystis* were homogenized and fed to 12 dogs (Heydorn and Rommel, 1972). Dogs excreted sporocysts after a prepatent period of 9 or 10 days and patent periods of 57–71 days; sporocysts were 13.9–11.7 × 6.2–10.8 *μ*m. The sporocysts were in the lamina propria of small intestine; the same dogs were refed infected beef when the sporocysts were no longer detected in feces; dogs excreted sporocysts 10–12 days later after ingesting infected beef for the second time. In these studies, the parasite was called *Isospora bigemina* (Heydorn and Rommel, 1972).

Experimental infection of cattle with *Sarcocystis* was performed by Gestrich *et al*. (1975). The proposal to name this species *S. bovicanis* by Heydorn *et al*. (1975*b*) was primarily based on this experiment. This paper does not have an English summary; therefore, we provide details here.

Eight, 7- to 8-weeks-old calves were orally inoculated with 50 000 (1 calf), 100 000 (2 calves) or 2 000 000 (5 calves) sporocysts; the calves were necropsied on days 28 (2 calves), 29 (2 calves), 30 (1 calf), 48 (1 calf), 62 (1 calf) post inoculation (p.i.), and muscle biopsy were taken from 1 calf, on days 160 and 194 p.i. (Gestrich *et al*., 1975). All 5 calves inoculated with 2 000 000 sporocysts died of acute sarcocystosis; schizonts were found in blood vessels. Thin-walled (<0.5 *μ*m) sarcocysts were detected in muscles of calves examined days 48, 62 and 98 p.i.; no mention was made concerning days 160 and 194 p.i. (Gestrich *et al*., 1975).

Subsequently, development of *S. cruzi* sarcocyst was studied ultrastructurally by Heydorn *et al*. (1975*a*) and Mehlhorn *et al*. (1975) using 4 calves inoculated with 50 000–80 000 000 sporocysts. Details of inoculation, dose and necropsy are provided here for the benefit of readers who may not have access to these papers in German. Calf 1 was inoculated with 80 000 000 sporocysts and necropsied on day 27 p.i. Calf 2 was inoculated with 50 000 sporocysts, and reinoculated with 100 000 on 14 days later; the same calf received 250 000 sporocysts weekly for 7 weeks, and 2 000 000 on day 60 and then necropsied on day 150 from the initial inoculation of sporocysts (Mehlhorn *et al*., 1975). The third calf was inoculated with 100 000 sporocysts and with 2 000 000 sporocysts on day 42; the calf was necropsied on day 76 p.i. (day 34 after the second dose). The fourth calf received 100 000 sporocysts and was necropsied on day 62 p.i. Sarcocyst formation was reported beginning day 34 p.i. but the duration of infection remains unclear because the calf was inoculated several times. At day 62 p.i., sarcocysts contained mainly metrocytes and a few bradyzoites. Sarcocysts at day 76 p.i. contained bradyzoites. Details of sarcocyst development were given by Mehlhorn *et al*. (1975) and Heydorn *et al*. (1975*a*) described structures of bradyzoites (they called them merozoites); both studies are based on the same calves.

## Summary of research on *S. cruzi* infections in cattle at USDA

Pioneering research on bovine sarcocystosis was performed at the Animal Parasitology Institute (now Animal Parasitic Diseases Laboratory, APDL), United States Department of Agriculture (USDA) Beltsville, Maryland under the direction of Dr. Ronald Fayer (now retired); these studies are summarized here (Table S1). These studies established for the first time that *S. cruzi* is pathogenic for cattle. Calves orally inoculated with 250 000–1 000 000 sporocysts developed acute systemic disease and intravascular schizonts were found in almost all organs of calves (Fayer and Johnson, 1973). Cows inoculated with sporocysts from dogs aborted and developed acute sarcocystosis (Fayer *et al*., 1976*a*). Results of this experiment also linked the mysterious Dalmeny disease reported by Corner *et al*. (1963) to acute *S. cruzi* infection. Other studies on the biology of experimental *S. cruzi* infection are also summarized in Table S1.

Fayer and associates also described gametogony and sporogony of *S. cruzi* in intestines of dogs fed beef from experimentally or naturally infected calves and its transmission to different hosts (Fayer, 1974, 1977*a*; Fayer *et al*., 1976*b*; Sheffield and Fayer, 1980); these studies are discussed later.

In the 1980s, one of us (J. P. D.) studied the full life cycle of a *S. cruzi* transmissible from coyotes to cattle in Bozeman, Montana, USA. For the life cycle studies with the Bozeman isolates of *S. cruzi*, newborn calves and coyotes were raised in captivity to strictly monitor their diets (Dubey, 1982*a*) (Table S2).

While reevaluating histologic slides from the 1982 study (Dubey, 1982*a*), a paraffin block of a kidney of a calf 383 fed 150 000 000 *S. cruzi* sporocysts from coyote no. 27 (Table S2) and euthanized on day 24 p.i. was located, and DNA was extracted. Here, we report the first genetic characterization of *S. cruzi* from this experimentally infected kidney.

## Materials and methods

### Specimens studied

In view of the recent development and recognition of several *Sarcocystis* species in cattle, we reevaluated histologic sections and obtained new morphological and genetic data from parasites used by Dubey (1982*a*). Life cycle stages were photographed using a digital DP73 camera (Olympus Optical Ltd., Tokyo, Japan) fitted on an Olympus AX 70 microscope (Olympus Optical Ltd.), and deposited in the Smithsonian Museum.

### Inoculation of calves at Bozeman

Materials and methods were the same as described in detail previously (Dubey, 1982*a*). Briefly, 38 Holstein–Friesian newborn calves were inoculated with 275 000–500 000 000 sporocysts of 7 isolates (BI–B7) of *S. cruzi* obtained from laboratory-raised coyotes fed naturally infected hearts of cattle raised in Montana (Tables S2 and S3). Calves were necropsied between days 4 and 153 p.i. (Table S2). Parasitaemia was determined as described elsewhere (Dubey, 1982*c*).

A total of 19 coyotes were used in this study and they were obtained from dens within a week of birth and were raised on canned milk and canned or dry dog food (Dubey, 1982*c*).

Four coyotes were necropsied for histological studies. For the study of early stages of gametogenesis, one coyote was killed at 6 h and another at 12 h after ingesting a single meal of infected meat. A third coyote was fed 2 meals, 6 h apart, of infected meat and killed 18 h after ingesting the last meal. The infected meat consisted of tongue, heart, oesophagus and other skeletal muscles of experimentally inoculated calf 422 (Table S3). A fourth coyote was euthanized 16 days after feeding experimentally infected beef (Table S3).

To assess the potential of the coyote as definitive host of *S. cruzi* in the wild, 15 coyotes fed infected beef (Table S3) were euthanized, the small intestine mucosa and the submucosa was scraped from the muscular layers, homogenized in a blender and sporocysts were collected as described before (Dubey, 1980). Additionally, sporocysts were collected from sugar flotation of the entire daily feces of each coyote (Table S3).

### Transmission of dog-transmitted *S. cruzi* infected with Beltsville strain to coyote and cattle

To study if the Beltsville strain of *S. cruzi* derived from a cattle–dog cycle was also transmissible to coyotes, beef from a calf inoculated with the dog-derived *S. cruzi* sporocysts was sent cold by air by Dr Fayer from Beltsville, Maryland to Bozeman, Montana where it was fed to a laboratory-raised coyote; the coyote started excreting sporocysts on day 9 p.i. The coyote was euthanized on day 12 p.i. and 50 000 000 sporocysts from the intestinal digest were fed to a newborn calf and the calf was euthanized on day 15 p.i.

At necropsy, virtually all internal organs of cattle were studied histologically as described (Dubey, 1982*a*). Portions of tissues were fixed in 10% (v/v) neutral buffered formalin. Similar procedures were followed for tissues of coyotes. Selected tissues were also fixed in Bouin's fluid (BF) or Helly's fixative. Paraffin-embedded sections were cut at 5 *μ*m. Selected tissues were embedded in glycol methacrylate (called plastic here) and sectioned at 3 *μ*m. Sections were stained with haematoxylin and eosin (HE), Heidenhain's iron haematoxylin (IH) or periodic acid-Schiff'shematoxylin (PASH). A similar process was used for tissues of coyotes. The histological slides evaluated were those from the 1980 study.

### Molecular characterization

Recently, we performed molecular characterization of parasites that had been embedded, 4 decades ago, in a paraffin block containing kidney tissue of experimentally infected calf no. 383 (Table S2). Genomic DNA extraction was performed using the Qiagen DNeasy® Blood and Tissue Kit and Qiagen QIAmp® DNA FFPE Tissue Kit according to the manufacturer's instructions and stored at −20°C for further use. The quality and quantity of the DNAs were calculated using a Qubit 4 Fluorometer (Invitrogen, ThermoFisher Scientific, Waltham, MA, USA).

For genotyping, *S. cruzi*-specific primers (18S rRNA, 28S rRNA, COX1 and ACS genes) were designed using Primer3 version 4.1.0 (Untergasser *et al*., 2012) and the National Center for Biotechnology Information (NCBI) primer blast using *S. cruzi* as reference sequence from NCBI (Table S4). The 25 *μ*L of polymerase chain reaction (PCR) mix consisted of a 2 *μ*L DNA template (120 ng *μ*L^−1^), 12.5 *μ*L of Platinum Hot Start PCR Master mix (Invitrogen, USA), 1 *μ*L of 10 pmol *μ*L^−1^ of each primer (IDT, USA) (Table S4) and 8.5 *μ*L of molecular grade water. Templates were denatured at 94°C for 3 min, followed by 35 cycles of denaturation at 94°C for 30 s, annealing at 60°C for 30 s and elongation at 68°C for 20 s min; these cycles were followed by a terminal elongation at 68°C for 5 min. The PCR products were analysed on a 2% agarose gel running at 100 V for 30 min and the size of the amplicons was estimated by comparison with the 100 bp DNA Ladder (Promega, USA).

The PCR products were purified using the ExoSAP method (Bell, 2018) and sequenced using the Big Dye Terminator v3.1 cycle sequencing kit (Applied Biosystems™, USA), following the manufacturer's instructions, in an ABI 3100 genetic analyser (Applied Biosystems™, USA). The sequences were deposited in the Genbank database and accession numbers were obtained (Table S4).

### Phylogenetic analysis

Phylogenetic analyses were performed independently on nucleotide sequences of 18 rRNA, 28S rRNA, COX1 and acetyl-CoA synthetase genes to evaluate the evolutionary relationship between *S. cruzi* and other closely related *Sarcocystis* spp. The segments obtained were trimmed and aligned by MAFT alignment in Geneious software followed by ClustalW multiple alignment in MEGA7 (Kumar *et al*., 2016). Any ambiguous bases were clarified using the respective chromatograms. Subsequently, the consensus sequence of the SSU rDNA was analysed using a standard online Basic Local Alignment Search Tool (BLAST) (https://blast.ncbi.nlm.nih.gov/Blast.cgi) against the genetic dataset of various *S. cruzi* species available at National Center for Biotechnology Information (NCBI). For generating consensus trees, we first blasted the edited sequences against the National Center for Biotechnology Information (NCBI) database and downloaded the most closely related *Sarcocystis* species. This allowed us to characterize a well-supported clade of *S. cruzi* to which our sequences belong. The sequences were subjected to phylogenetic analysis using *MEGA 7* software (Kumar *et al*., 2016). A codon-based ClustalW multiple alignment of all sequences was performed in MEGA X. Neighbor-Joining (NJ) trees were obtained. To assess the robustness of the phylogenetic trees, 1000 bootstrap replications were employed in all analyses. All codon positions were used.

The molecular analysis was performed against the existing partial sequences previously obtained from different isolates of *S. cruzi* from cattle, and 8 haplotypes from *Sarcocystis* species identified in Bovidae and Cervidae. All sequences were truncated slightly at both ends to preserve the homologous nucleotide positions for further analysis. *Neospora caninum* and *Toxoplasma gondii* were used as out-groups. For acetyl CoA synthetase gene, we downloaded the sole available haplotype (acc. no. LC729541) and found it identical to our isolate of *S. cruzi*.

## Results

The following description of the life cycle stages is from the coyote–cattle cycle of *S. cruzi* as reported previously (Dubey, 1980, 1982*a*, 1982*c*; Dubey *et al*., 1982), and in the present study.

### Intravascular stages

#### Terminology used

Different terms have been used to describe *S. cruzi* asexual stages. Historically, coccidian (*Eimeria* spp.) asexual stages are called schizonts (Dubey, 2020). In schizogony, the sporozoite nucleus divides into 4 or more nuclei (>1000 in *Eimeria bovis*) before merozoites are formed. However, in some other coccidians (e.g. *T. gondii*) some stages divide into 2 daughter organisms by endodyogeny. To encompass all coccidians, Levine (1973), an expert on coccidian parasites, called asexual stages as meronts instead of schizonts. Dubey (1982*a*) followed Levine and called *S. cruzi* intravascular stages as meronts. However, other authors (Fayer and Johnson, 1973; Gestrich *et al*., 1975) called them schizonts. For uniformity, we have now used the term schizonts for *S. cruzi*. We are aware of the differences in asexual cycle in *Eimeria vs Sarcocystis*. Unlike *Eimeria*, *S. cruzi* schizonts divide by endopolygeny, where the parasite nucleus becomes multilobed but remains interconnected (Dubey *et al*., 2016).

**Figure 1.**
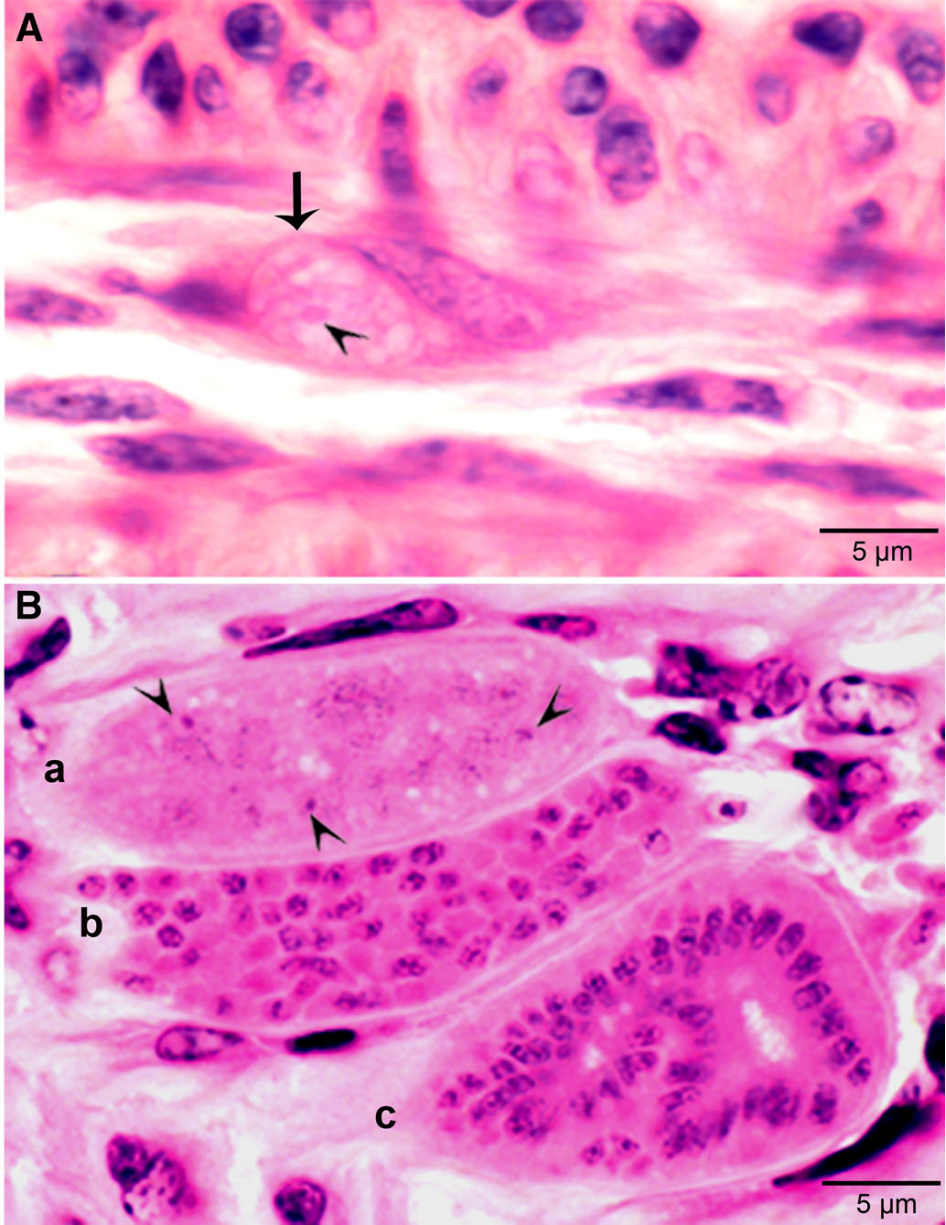
Development of first generation schizonts of *Sarcocystis cruzi* in arteries of calves. (A) Longitudinal section of an artery in mesenteric lymph node of a calf 36, day 11 p.i. Note an early schizont (arrow). The infected endothelial cell is protruding in the lumen. Note the central nucleus (arrowhead) of the schizont. HE. (B) Cross-section of a renal artery showing 3 schizonts (a, b, c) in sequential stages of development. The schizonts have occluded the lumen of the artery. Plastic-embedded 3 *μ*m section. HE. (a) Early immature schizont with lobulation of nucleus with nucleoli (arrowheads). (b) Maturing elongated schizont with merozoite formation, each nuclear lobe is incorporated in a merozoite. (c) An almost mature schizont with radially arranged merozoites.

In *S. cruzi*, at least 3 generations of intravascular stages were identified. To trace the development of earliest stages, 2 calves each fed 500 000 000 sporocysts were euthanized on day 4 and 7. No development was found earlier than day 11 p.i. At day 7 p.i., sporozoite-like zoites were found within intravascular leucocytes and in endothelial cells of arteries within mesenteric lymph nodes. After an intensive search, a total of 36 zoites were found in numerous histological sections of the mesenteric arteries; 31 of these were in endothelial cells, 4 were in macrophage-like leucocytes in vascular lumen and 1 was apparently extracellular (Dubey, 1983). Immature schizonts were found in mesenteric lymph node arteries on day 11 p.i. (Fig. 1A) and mature first generation schizonts were found on days 15–26 p.i. (Fig. 1B). The development of schizonts was asynchronous. Schizonts averaged 41.0 × 17.5 *μ*m and contained up to 350 merozoites.

**Figure 2.**
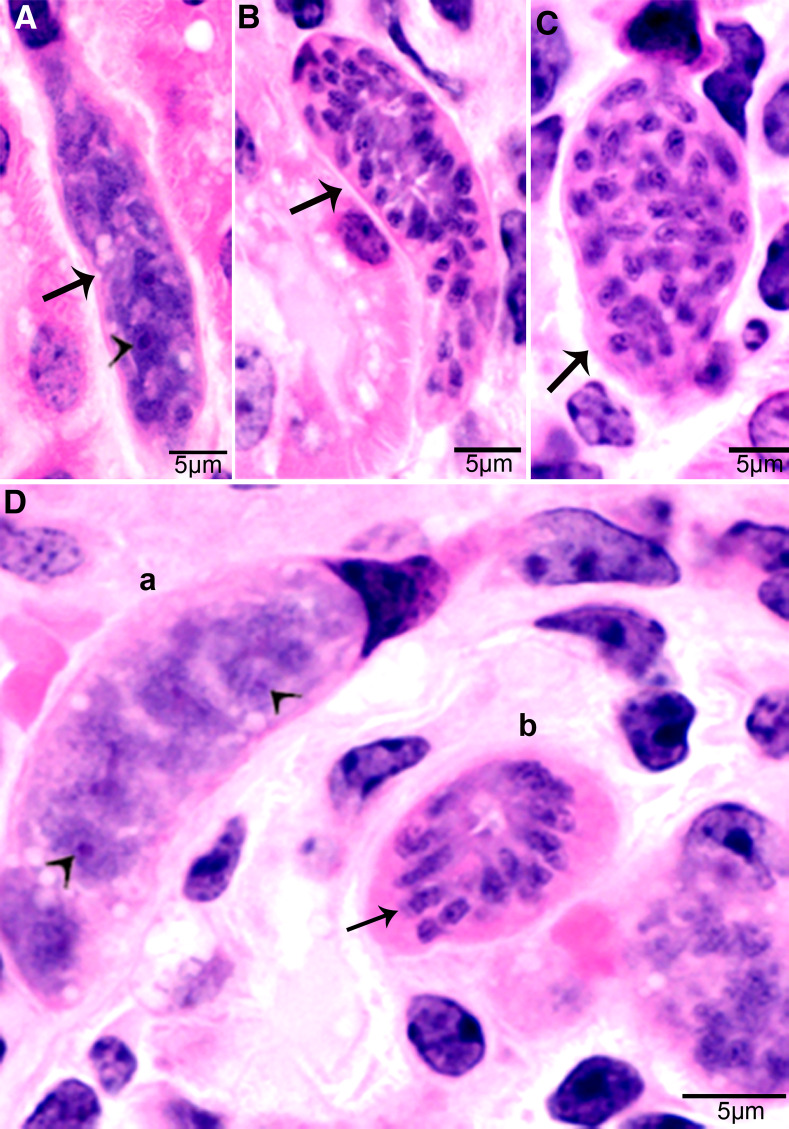
Development of second-generation *Sarcocystis cruzi* schizonts in kidney of calf 383, day 24 p.i. Plastic-embedded 3 *μ*m section. HE. (A) Early immature narrow multilobed schizont (arrow) in interstitium; note prominent nucleoli (arrowhead). (B) Immature schizont (arrow) in glomerulus with nuclear lobes separated by clefts. (C) Schizont (arrow) with randomly arranged nuclear lobes. (D) Two schizonts in glomerulus. (a) Immature schizont (arrow) in glomerulus with nucleoli (arrowhead) in nuclear lobes. (b) A mature schizont with radially arranged merozoites (arrow).

Second generation schizonts were found in capillary endothelial cells of many organs but were most numerous in kidneys on days 19–46 p.i. (Fig. 2). The shape and size of schizonts varied, depending on the tissue parasitized; renal schizonts (19.6 × 11.0 *μ*m) were shorter than intramuscular schizonts (25.0 × 11.0 *μ*m).

### Parasitaemia and multiplication in blood

**Figure 3.**
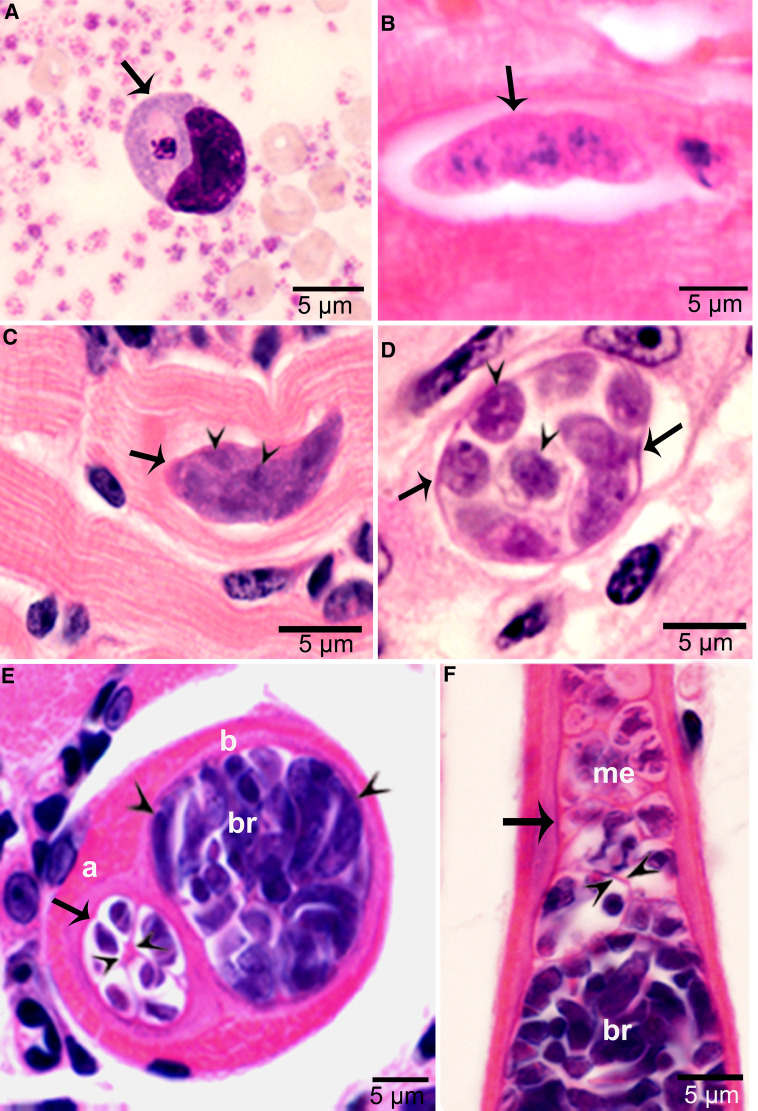
Asexual stages of *Sarcocystis cruzi*. (A) Merozoite in buffy coat smear of peripheral blood of calf 412, day 28 p.i. For size comparison, erythrocytes and thrombocytes are presented. Giemsa stain. (B) Early sarcocyst (arrow) in parasitophorous vacuole (PV) of myocardium of calf 601, day 42 p.i. HE. (C) An immature sarcocyst (arrow) with 6 metrocytes (arrowheads) in PV of diaphragm of calf 460, day 55 p.i. HE. (D) Cross-section of an immature sarcocyst in myocardium of calf 460, day 55 p.i. Plastic-embedded 3 *μ*m section. HE. Note sarcocyst wall (arrows). The metrocytes (arrowheads) are globular. (E, F) Sarcocysts in tongue of calf 488, day 153 p.i. HE. Sarcocysts have thin walls (arrow). The eosinophilic area surrounding the sarcocysts is degenerated host tissue giving false impression that the cysts are thick walled. (E) Cross-section of 2 sarcocysts (a, b), apparently within 1 myocyte. The sarcocyst on the left is immature and contained metrocytes are separated by septa (arrowheads). (F) Longitudinal section of a sarcocyst at its one end. Note intensely stained bradyzoites (br) and lightly stained metrocytes (me), and thin septa (arrowheads).

Two waves of parasitaemia were recognized. Few merozoites were found in blood on day 17 p.i., coincident to maturation of first generation schizonts. The second wave of parasitaemia occurred on days 24–46 p.i. Most (97.2%) merozoites were within lymphocytes or monocytes (Fig. 3A). The number of merozoites ranged from 0.14 to 9.8 per mL of peripheral blood (Dubey, 1982*c*). A few merozoites divided in monocytes, apparently by endodyogeny (division of into 2 nuclei). All dividing merozoites were intracellular; these merozoites were 6.8 × 3.2 *μ*m in Giemsa-stained smears of blood.

**Figure 4.**
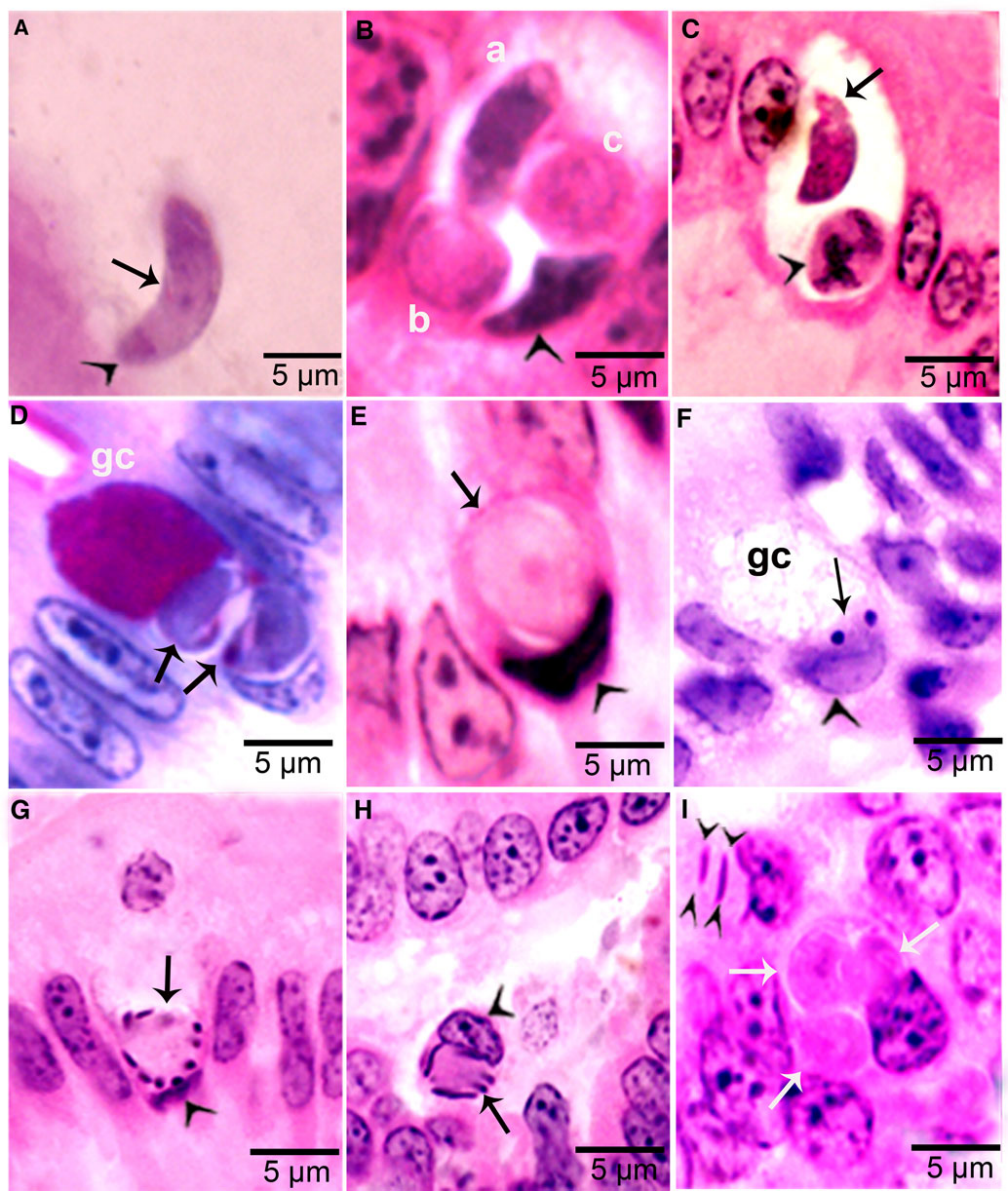
Development of sexual stages of *Sarcocystis cruzi* in sections of small intestine of coyotes. The epithelial brush border is oriented up towards the intestinal lumen in B–G. (A) Bradyzoite (arrow) in intestinal lumen. The apical end is stained intensely (arrowhead) and the nucleus (arrow) is central. Plastic-embedded 3 *μ*m section. Coyote 34, 6 h p.i. HE. (B) Three organisms (a, b, c) in a goblet cell. The zoite in (a) is probably a bradyzoite and (b, c) are macrogamonts. Arrowhead points to host cell nucleus. Coyote 34, 6 h. HE. (C). Two organisms in a goblet cell. The organism marked by arrow is probably a bradyzoite and the one marked by arrowhead is a macrogamont with dividing nucleus. Coyote 44, 12 h p.i. (D) Two macrogamonts within a goblet cell (gc) intensely stained red with PASH. Coyote 34, 6 h p.i. (E) A macrogamont with central nucleus. Arrowhead points to host cell nucleus. Coyote 34, 6 h p.i (F) A binucleate microgamont (arrow) in a goblet cell (gc). Arrowhead points to host cell nucleus. Coyote 34, 6 h p.i. (G) A microgamont with gametes at the periphery. Arrowhead points to host cell nucleus. Coyote 34, 6 h p.i. (H) Mature microgamont (arrow) in the lamina propria. Coyote 44, 12 h p.i. (I) Two microgametes (opposing arrowheads) apparently free in a cell. Also note 3 macrogamonts (white arrows). Coyote 44, 12 h p.i.

Sarcocysts were found within myocytes, most commonly in the heart and tongue. The earliest sarcocysts were found on day 42 p.i. and contained 1 or 2 metrocytes. At day 46 p.i., sarcocysts were mostly in the myocardium and contained up to 8 metrocytes. At day 55 p.i., sarcocysts were <20 *μ*m and contained only metrocytes (Fig. 3C and D). Metrocytes were round to elongate, and usually stained faintly with HE stain. At day 67 p.i., sarcocysts were up to 360 *μ*m long and 30 *μ*m wide; they had only metrocytes. At day 86 p.i., sarcocysts contained both metrocytes and bradyzoites and were infectious for coyotes. The sarcocyst wall was thin (<l *μ*m) and smooth. Sarcocysts remained microscopic in all calves, including the last day of observation, day 153 p.i. (Fig. 3E and F). Bradyzoites were 11 × 3.0 *μ*m in smears and 8.2 × 3.0 *μ*m in sections (Fig. 4A). Degenerating sarcocysts were not found.

Only a few sarcocysts were found in brain and spinal cord tissues. Immature sarcocysts containing metrocytes were found in smooth muscles of the small intestine, urinary bladder, rumen and abomasum (Dubey, 1982*a*).

Only 1 morphologic type of sarcocysts, corresponding to *S. cruzi*, was found in calves inoculated with sporocysts from coyotes and the development correlated with day p.i. The identity of *S. cruzi* sarcocysts was confirmed ultrastructurally (Dubey, 1982*a*).

### Gametogony

Gamonts were seen in parasitophorous vacuoles (PVs) of goblet cells in the epithelium of the small intestine at 6 h p.i. (Fig. 4B and C). The host cell nucleus was indented, misoriented, but not hypertrophied (Fig. 4B, D, E and G). Numerous free bradyzoites were seen in contents of stomach, small intestine and colon (Fig. 4A). At 6 h, gamonts were found throughout the tips of the villi of the small intestine of the coyote. Most of the round to elongate gamonts were found in goblet cells; up to 3 gamonts were seen in a single goblet cell (Fig. 4B). A few gamonts were observed in the lamina propria just below the basement membrane (Fig. 4H). Fewer than 5% of gamonts were microgamonts. All stages of microgametogony, from the single nucleated stage to fully developed gametes, were seen (Fig. 4F–I). Mature microgamonts were 7.1 × 4.8 *μ*m and contained 3–11 slender gametes, which were 3.5–4.0 *μ*m long and less than 0.5 *μ*m wide (Fig. 4I). Macrogamonts were 8.0 × 7.3 *μ*m and contained PAS-positive granules.

**Figure 5.**
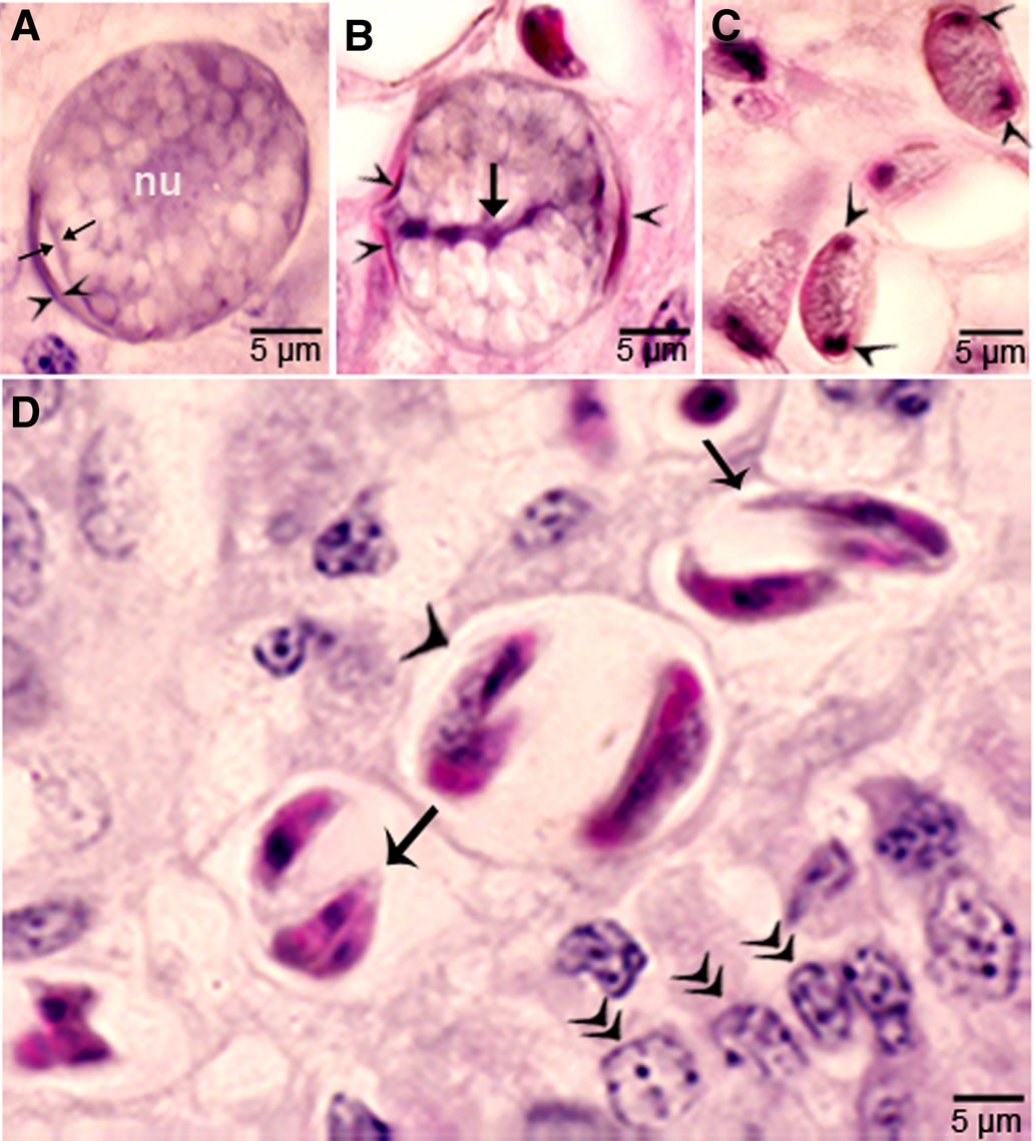
Sporogony of *Sarcocystis cruzi* in small intestine of a coyote 43, day 16 after ingesting infected beef. Plastic-embedded 3 *μ*m section. HE. (A) Unsporulated oocyst with a central nucleus (nu). The outer layer of oocyst wall is partly formed (opposing arrowhead). The inner layer is thin (arrow). (B) Unsporulated oocyst. The outer layer of oocyst wall is partly formed (arrowhead). The inner layer is thin (opposing arrows). Note a central band of basophilic nucleus (arrow). (C) Sporocysts with polar nuclei (arrowheads). (D) Sporulated oocysts/sporocysts (arrowheads) in the lamina propria, beneath the epithelium (double arrowheads). Note sporozoites (arrow).

At 12 h, stages resembled those at 6 h. Additionally, 2 oocyst-like structures were seen in Giemsa-stained smears of the intestine. By 24 h, most gamonts had become oocysts. In the coyote killed on day 16 p.i., only oocysts were found; they were in the lamina propria (Fig. 5 A–D). Unsporulated oocysts contained a central or a band nucleus (Fig. 5B). Oocysts grew to about 20 × 19 *μ*m before sporulation began. All stages of sporulation, from binucleated sporocysts to fully sporulated sporocysts, were seen on day 16 p.i. (Fig. 5D). Living sporozoites were around 10 × 3 *μ*m whereas those in sections were around 9 × 2 *μ*m.

The prepatent period was 9 days in all 16 coyotes (9 coyotes fed naturally infected beef, 7 coyotes fed experimentally infected beef, including 1 coyote fed the beef infected with the Beltsville strain) (Table S3).

### Transmission of dog-transmitted *S. cruzi* infected with Beltsville strain to coyote and cattle in Bozeman

Coyote no. 30 fed beef infected with the Beltsville strain of *S. cruzi* started excreting sporocysts on day 9 p.i. and 300 000 000 sporocysts were recovered in intestinal digest when the coyote was euthanized (Table S3). The newborn calf orally inoculated with 50 000 000 sporocysts remained asymptomatic. Many first generation schizonts were found in histological sections of arteries in kidneys and mesenteric lymph nodes when the calf was euthanized on day 15 p.i. These schizonts were structurally similar to those described by Fayer (1977*b*).

### Molecular characterization

**Figure 6.**
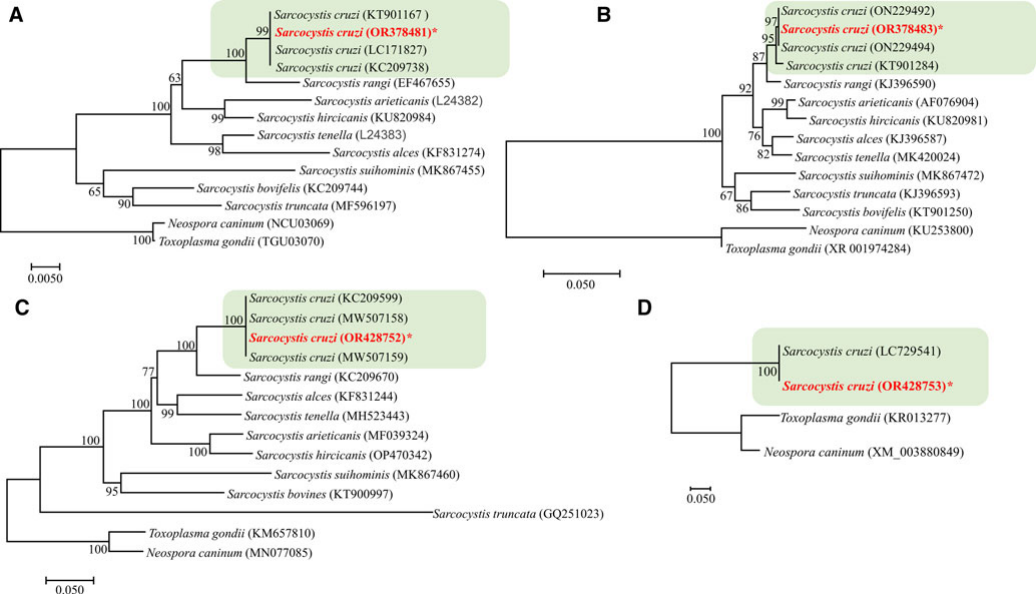
Phylogenetic relationships of *Sarcocystis cruzi* with selected members of the Sarcocystidae inferred from various genetic markers (A = 18S rRNA, B = 28S rRNA, C = *COX1*, D = Acetyl CoA genes *ACS*) under Neighbor-Joining criterion (1000 bootstrap values; p-distance mode l; pairwise deletion). Branch supports are indicated near the corresponding nodes. Asterias is indicated against the species under study and the highlighted part shows the *S. cruzi* cluster with highest nodal support.

The sequencing of *S. cruzi* from the kidney of experimentally infected calf resulted in sequences of 1391 bp for 18S rRNA gene; 583 bp for 28S rRNA; 960 bp for COX1 and 1743 bp for ACS gene with a guanine–cytosine–GC content of 41.6, 43.2, 51.9 and 54.8, respectively. In the phylogenetic analyses conducted using the NJ method based on partial 18S rRNA gene sequences (Fig. 6), the placement of the 2 major clades comprising *Sarcocystis* spp. of cattle showed some variation depending on the sequence variability and alignment settings, particularly the gap opening and gap extension penalties.

BLAST analysis showed 99–100% similarity of the present isolate to other available isolates attributed to *S. cruzi* from naturally infected cattle. Phylogenetic reconstruction of variation in 18S rRNA sequences, using the NJ method, confirmed its uniquely close relationship to other *S. cruzi* isolates, to the exclusion of other congeners (Fig. 6). The newly sequenced isolate belongs to a group comprised only of other isolates of *S. cruzi*, with strong bootstrap support, and distinct from sequences from other parasites of cattle including *S. rangi*, *S. arieticanis*, *S. hircicanis*, *S. tenella*, *S. alces*, *S. suihominis*, *S. bovifelis* and *S. truncata* (Fig. 6). Relationships among species generally accord with prior findings (Gjerde, 2013, 2016).

### Bison (*Bison bison*) as an intermediate host of *S. cruzi*

Bison can act as intermediate host of *S. cruzi*. A laboratory-raised coyote (no.13) excreted sporocysts 11 days after feeding bison meat in Montana (Dubey, 1980). Three cattle calves inoculated with 100 000 (1 calf) and 700 000 (2 calves) sporocysts from coyote 13 developed patent *Sarcocystis* infections (Fayer *et al*., 1982). Two calves had diarrhoea on days 19–25 p.i. One of the 2 calves fed 700 000 sporocysts became moribund on day 28 p.i. and was necropsied. Haemorrhages were found in many organs, particularly on the cecum; there is no record of histological examination of tissues in the paper and these specimens are no longer available for retrospective evaluation. Sarcocysts characteristic of *S. cruzi* were found in histological sections of the 2 calves necropsied on days 63 and 89 p.i. (Fayer *et al*., 1982).

For experimental transmission of the cattle strain of *S. cruzi*, 2 newborn laboratory-raised bison were inoculated orally with sporocysts of B1 isolate of *S. cruzi* originally isolated from a laboratory-raised coyote (coyote 25, see Table S3) fed naturally infected beef in Montana. A second passage of this isolate was used to inoculate each bison. For this, sporocysts from coyote 25 were fed to a newborn cattle calf and muscles from the calf were fed to laboratory-raised coyotes (nos. 38 and 43, see Table S3). Sporocysts from coyotes 38 and 43 were used as inocula for bison (Dubey, 1982*b*). One 6-day-old bison was fed 10 000 000 sporocysts. This bison developed transient diarrhoea and fever on day 14 p.i. then remained asymptomatic until day 25 p.i. The bison was euthanized on day 28 p.i. because it had severe diarrhoea and was unable to swallow. Second generation schizonts were found in histological sections of the adrena gland cortex and in the lamina propria of small and large intestines. Merozoites were detected in peripheral blood on days 25–28 p.i.

The second bison was inoculated with 100 000 sporocysts of the B1 isolate when 7 days old; this bison had fever on days 16 and 18 and then again on days 27–30 p.i. (Dubey, 1982*b*). Sarcocysts were found in muscles when the bison was necropsied day 73 p.i. Merozoites, schizonts and sarcocysts found in the bison tissues were like those of *S. cruzi* in cattle tissues. The unusual findings regarding bison infections were: diarrhoea and the site of second generation schizonts; in cattle, the kidney is the preferential site for second generation schizonts, whereas in the bison, intestines and adrenal glands were the parasitized sites. Diarrhoea is not a typical clinical feature of acute sarcocystosis in cattle.

In summary, *S. cruzi* was transmissible from bison to cattle and cattle to bison *via* coyotes. The bison isolate of *S. cruzi* was apparently more pathogenic than the cattle strains and its schizonts were in the intestines than in the kidney most often parasitized by cattle strains. Nothing is known of the genetic features that define strain differences in *S. cruzi.*

### Taxonomic summary of *S. cruzi*

*Diagnosis:*

*Intermediate host:* Cattle (*Bos* spp.), Bison (*Bison* spp.)

Asexual development in bovine tissues. Two morphologically distinct generations of schizonts directly in endothelial cells of blood vessels, particularly in kidneys 11–46 days p.i. followed by parasitaemia and development of merozoites in peripheral blood monocytes. Sarcocysts in PVs of myocytes, particularly in the myocardium. Sarcocysts microscopic, <1 mm long, with thin (<0.5 um thick) sarcocyst wall.

*Definitive hosts*: Domestic dog (*Canis domesticus*), coyote (*Canis latrans*), red fox (*Vulpes vulpes*) confirmed.

Gamonts in epithelium, primarily in goblet cells of small intestine. Sporogony in the lamina propria of small intestine. Prepatent period 9 days or more. Sporulated sporocysts ~16 × 10 *μ*m.

### Specimens deposited

The specimens were deposited in United States National Parasite Collection in the Division of Invertebrate Zoology and National Museum of Natural History, Smithsonian Institution, Museum Support Center, MRC 534, 4210 Silver Hill Road, Suitland, Maryland 20746, USA, under numbers USNM 1606783-16067795 (Table 1).

**Table 1.** Details of *Sarcocystis cruzi* (Bozeman isolates) specimens deposited in the Smithsonian Museum

Specimen no.	Host	Histology case no.	Days (d) or hours (h) p.i.	Tissue	Figure no.	Stain	Museum no.
1	Calf 36	19252	11 d	Mesenteric lymph node	1A	HE	1606783
2	Calf 38	19253	15 d	Kidney	1B	HE	1606784
3	Calf 383	451-80	24 d	Kidney	2A, B, C	IH	1606785
4	Calf 412	None	28 d	Blood	3A	Giemsa	1606786
5	Calf 601	469-81	42 d	Heart	3B	IH	1606787
6	Calf 460	171-81	55 d	Diaphragm	3C	HE	1606788
7	Calf 460	171-81	55 d	Heart	3D	HE	1606789
8	Calf 488	414-81	153 d	Tongue	3E, F	HE	1606790
9A	Coyote 34	C-177-81	6 h	Small intestine	4A, B, E, F, G	HE	1606791
9B	Coyote 34	C-177-81	6 h	Small intestine	4D	PASH	1606792
10	Coyote 44	C-178-81	12 h	Small intestine	4C, H, I	HE	1606793
11	Coyote 43	C-190-81	16 d	Small intestine	5A–D	HE	1606794
12	Calf 383	451-80	24 d	Kidney	Paraffin block	None	1606795

DNA sequences were deposited in Genbank (Table S4).

## Discussion

### Intravascular stages

In general, the intravascular stages of *S. cruzi* found in dogs fed the Beltsville strain resemble those reported to have developed in coyotes fed infected beef of the Bozeman strains. The minor differences observed likely relate to dose, histological methods, time spent searching for early stages and age of calves. For example, only a few first generation schizonts were found in calves infected at Beltsville and these were immature (Fayer, 1977*b*). Using the Beltsville strain cycled through a coyote, numerous first generation schizonts were detected in arteries in kidneys and mesenteric lymph nodes and schizonts contained fully developed merozoites; these observations are probably related to the age of calves and dose; the calves were 3–4.5 months old and inoculated with 200 000 sporocysts at Beltsville, whereas a newborn calf was inoculated with 50 000 000 sporocysts at Bozeman.

Here, we summarized development of the ox–coyote cycle of *S. cruzi*. After the sporocysts excyst in cattle gut, the sporozoites reach arteries in the mesenteric lymph nodes, probably *via* portal circulation. The zoite found in blood on day 4 p.i. was probably a sporozoite, as were those found on day 7 p.i. The first generation meronts have a predilection for arteries, whereas second generation meronts occur predominantly in capillaries. Both first and second generation meronts develop in cells between the tunica intima and the endothelium (Dubey *et al*., 1980).

Second generation schizonts were first detected on day 19 p.i. and some had matured by day 24 p.i. This indicates a generation time of 5 or more days. Most of the second generation schizonts were found between days 24 and 29 p.i. Whether more than 1 multiplication cycle of second generation schizonts occurs was not determined.

Intramuscular schizonts were longer than renal meronts. Whether this phenomenon was related to the stage of development or to the type of host cell parasitized was not determined. Such elongated meronts were not reported by Fayer and Johnson (1973). In addition, individual merozoites were found in muscles on days 33–45 p.i. in the present study; these were not reported to occur in the calves examined by Fayer and Johnson (1973), who found individual merozoites in the kidneys.

### Sarcocysts

Sarcocysts are formed around the sixth week after inoculation of sporocysts. Dubey (1982*a*) did not find sarcocysts until day 45 p.i. After reevaluation of histological sections 35–44 days p.i., a few sarcocysts were detected on days 42 and 44 p.i (present study). Fayer and Johnson (1973) found sarcocysts containing up to 50 zoites in a calf killed on day 33 p.i.; it is likely that the sarcocyst observed were from a spontaneous infection acquired from eating contaminated hay. Distinction of sarcocysts from intramuscular schizonts is sometimes difficult unless sections are stained optimally. However, a PV around the zoites can distinguish them; sarcocysts are located in a PV whereas schizonts are located directly in the host cell cytoplasm without a PV. Structurally, second generation schizonts could be differentiated from early sarcocysts. The PAS-positivity is also a distinguishing feature. Schizonts are PAS-negative but zoites in sarcocysts are PAS-positive. However, PAS positivity of metrocytes in early sarcocysts varies, depending on fixation; BF was better than formalin for preserving PAS-positive polysaccharide granules.

Mehlhorn *et al*. (1975) also studied the development of sarcocysts. Beginning day 34 p.i., only sarcocysts were found and the sarcocyst wall remained thinner than 1 *μ*m even at day 150 p.i. Sarcocysts at day 76 p.i. were structurally mature but the infectivity to calves was not tested (Mehlhorn *et al*., 1975). Fayer and Johnson (1974) stated that the sarcocysts on day 54 p.i. contained only metrocytes; no mention was made when bradyzoites developed or when sarcocysts were infectious for dogs. Sarcocysts became infectious between days 67 and 86 p.i. in calves fed sporocysts from coyotes (Dubey, 1982*a*).

### Gametogony and sporogony

Gametogony was completed in the coyote intestine between 6 and 12 h p.i. It is likely that gamonts are formed earlier. It is noteworthy that bradyzoites convert to gamonts in goblet cells while still in the surface epithelium. Subsequent development occurs in unidentified cells in the lamina propria. How *S. cruzi* gamonts reach the lamina propria is unknown. Initial gamonts are intracellular in PV, mostly in goblet cells. One hypothesis is that the infected host cells drop down to the lamina propria. Fayer (1974) and Sheffield and Fayer (1980) first described the gametogony and sporogony of *S. cruzi* in the small intestine of dogs fed infected beef. By using transmission electron microscopy, Sheffield and Fayer (1980) documented fertilization of gametes and the formation of the oocyst wall beginning at 12 h. They also mentioned that gametes were extracellular within the lamina propria but provided no quantitative data. Female gametes contain polysaccharide granules and if the fertilized gametes are indeed extracellular then these likely provide energy for sporulation and survival in the environment. There are no ultrastructural studies on the sporulation of *Sarcocystis* oocysts. However, Fayer (1974) described sporulation of *S. cruzi* in intestines of dogs by light microscopy. Macrogamonts were detected on day 2 through 13 p.i. He found small PAS-positive granules in macrogamonts on day 5 and these grew in size and number until day 13. Sporulating oocysts were seen on days 7–13 p.i. On day 8 p.i., a nuclear band was seen across the centre of some oocysts and binucleate sporocysts were identified on day 8 p.i. The presence of this nuclear band is confirmed here (Fig. 5B). Sporulated oocysts/sporocysts were first detected on day 9 p.i. (Fayer, 1974).

### Prepatent and patent periods

As stated earlier, Heydorn and Rommel (1972) reported that dogs fed naturally infected beef excreted *S. cruzi* sporocysts 9 or 10 days later. Fayer (1974, 1977*a*) evaluated this topic in detail. He reported prepatent periods of 9–33 days, irrespective of whether the beef was from experimental or naturally exposed infections. Most dogs excreted sporocysts between days 11 and 16 p.i. The dogs excreted sporocysts intermittently up to 60 days p.i. (Fayer, 1977*a*). After feeding 500 g of naturally infected beef, 11 dogs excreted a total of between 861 000 and 3 764 000 sporocysts. More sporocysts were excreted by dogs after ingesting experimentally infected beef than naturally exposed beef (Fayer, 1977*a*). In 1 dog, as many as 20 077 000 sporocysts were excreted (Fayer, 1977*a*).

*Sarcocystis* infections in beef are common (Dubey *et al*., 2016). One of the explanations is that dogs are common on cattle farms. We are not aware of any surveys for the presence of *S. cruzi* sporocysts in feces of farm dogs. In a study of dogs from a Humane Center in Ohio, USA, *Sarcocystis* spp. sporocysts were found in 1.8% of 500 dogs; most of these dogs were probably domestic pets (Streitel and Dubey, 1976).

In contrast to these findings in dogs, all 16 coyotes (9 fed naturally infected beef and 7 fed experimentally infected beef) started excreting sporocysts on day 9 p.i. (Table S3). Thus, the minimum prepatent period is 9 days (not 8 days mistakenly reported in Dubey [1982*a*]). In the coyote–cattle study at Bozeman, patent period was not determined because coyotes were euthanized on days 12–27 p.i. and sporocysts were collected from the digest of entire small intestines. As many as 265 000 000 sporocysts were recovered from coyotes fed naturally infected beef and 300 000 000 sporocysts were present in a coyote fed experimentally infected beef (Table S3). Differences in these studies may be related to the dose, method of sporocyst detection and duration of observation period. For the study in coyotes, the entire daily feces were sugar-floated and tested microscopically. Additionally at necropsy, the entire small intestine was homogenized and digested to recover sporocysts. *Sarcocystis* sporocysts are trapped in the lamina propria and are released in the intestinal tract intermittently. No inflammatory response to sporocysts in the lamina propria was observed.

The 9-day prepatent period coincides with the completion of sporulation on day 9 (Fayer, 1977*a*). However, sporogony is asynchronous because at day 16 p.i., unsporulated oocysts were observed in the lamina propria of infected coyotes (Fig. 5). Only sporulated oocysts and free sporocysts are found in feces of dogs or coyotes. Thus, transport of oocysts from the lamina propria to the intestinal lumen is likely regulated by the parasite because no sporocysts were detected in feces until day 9 p.i.

In cattle and coyotes infected at Bozeman, only thin-walled *S. cruzi* sarcocysts were found; no other coccidian oocysts were detected in the feces of these coyotes (Dubey, 1982*a*). The unsporulated oocysts seen by Fayer (1974) and Heydorn and Rommel (1972) in feces of dogs fed *Sarcocystis*-infected beef belong to another coccidian, *Hammondia heydorni*; it cycles through ruminants and canids (Dubey *et al*., 2002).

### Definitive hosts of *S. cruzi*

The domestic dog, coyote and red fox are proven definitive hosts of *S. cruzi* because sporocysts from these hosts were infectious to cattle, and infected beef proved infectious to laboratory raised dog, coyote and fox (Table 2). There is 1 report of a crab-eating fox from Brazil as a definitive host for *S. cruzi*, but it needs confirmation. Wolves and raccoon dogs fed naturally infected beef also excreted *S. cruzi* – like sporocysts, but infectivity of wolf or raccoon-derived sporocysts has not been tested (Table 2). Adult raccoons excreted *S. cruzi*-like sporocysts after ingesting *S. cruzi*-infected beef; however, the raccoons were adults when trapped (Fayer *et al*., 1976*b*). Subsequently, 2 studies could not confirm these findings (Table 2). Therefore, we do not consider raccoons as an established definitive host of *S. cruzi*. Although jackal (*Canis aureus*) was mentioned as a definitive host for *S. cruzi* (Dubey and Rosenthal, 2023) and sporocysts have been found in feces of naturally infected jackals (Kirkova *et al*., 2011; Tulov, 2013; Gherman and Mihalca, 2017), there is no evidence that the species was *S. cruzi.*

**Table 2.** Definitive hosts of *Sarcocystis cruzi* and development of gametogony

Definitive host	Country	Inoculum	Prepatent period (days)	Patent period	Gametogony	Infectivity from sporocysts to calves	Reference
Dog (*Canis familiaris*)	Germany	Naturally infected beef	9 or 10	70 days	Not studied, oocysts in lamina propria	Yes	Heydorn and Rommel (1972)
Dog (*Canis familiaris*)	USA	Naturally infected beef	9–22	3–16 days	Sporogony in lamina propria	Yes	Fayer (1974)
Dog (*Canis familiaris*)	USA	Experimentally infected beef			Gametogony	Yes	Sheffield and Fayer (1980)
Coyote (*Canis latrans)*	USA	Feces of naturally infected coyotes fed to calves^a^				Yes	Fayer and Johnson (1975)
Coyote (*Canis latrans)*	USA	Experimentally infected beef	9 days	Not studied	Gametogony and sporogony in lamina propria	Yes	Dubey (1982*a*)
Wolf (*Canis lupus*)	Germany	Naturally infected beef	9 or 10	>6 weeks	Not studied	Not tested	Rommel *et al*. (1974)
Red fox (*Vulpes vulpes*)	Germany	Naturally infected beef	9 or 10	>6 weeks	Not studied	Not tested	Rommel *et al*. (1974)
Red fox (*Vulpes vulpes*)-subspecies *V. fulva*	USA	Naturally infected beef	11		Not studied	Not tested	Fayer *et al*. (1976*b*)
Red fox (*Vulpes vulpes*)	USA	Experimentally infected beef	9			Yes	Dubey (1983)
Raccoon (*Procyon lotor*)^b^	USA	Naturally infected beef	8	Not studied	Not studied	Not tested	Fayer *et al*. (1976*b*)
Raccoon (*Procyon lotor*)	Germany	Experimentally infected beef	No sporocysts			Not tested	Heydorn and Matuschka (1981)
Raccoon (*Procyon lotor*)	USA	Experimentally infected beef	No sporocysts			Not tested	Dubey and Blagburn (1983)
Crab-eating fox (*Cerdocyon thous*)	Brazil	Naturally infected feces infective to a calf, muscles from calf infective to a dog					Rodrigues *et al*. (2008)
Raccoon dog (*Nyctereutes procyonoides viverrinus*)	Japan	Naturally infected beef	9	>6 weeks	Not studied		Saito and Itagaki (1994)

a Two calves fed sporocysts (135 000 or 720 000) from feces of naturally infected coyotes from Utah developed acute sarcocystosis.

b Raccoons were adults when trapped.

Recently, mustelids in Lithuania were reported as possible definitive hosts for 4 *Sarcocystis* species infecting cattle (*S. cruzi*, *S. bovifelis*, *S. hominis*, *S. hirsuta*). DNA was obtained from sporocysts separated from intestinal scrapings of mustelids and characterized by nested PCR of partial COX1 gene (Prakas *et al*., 2021). High detection rates of 2 species, *S. bovifelis* (89.3%) and *S. cruzi* (73.8%) were observed, while *S. hirsuta* (3.6%) and *S. hominis* (1.2%) have been found in only a few animals. The DNA of *S. cruzi* was identified in 77.5% of 40 American mink (*Neovison vison*), 75% of 4 Beech marten (*Martes foina*), 75% of 20 European pine marten (*Martes martes*), 20% of 5 European badger (*Meles meles*) and 80% of 15 European polecat (*Mustela putorius*). However, none of these carnivores were previously identified as definitive hosts for *Sarcocystis* species of cattle in other countries (Dubey *et al*., 2016).

There are only a few reports of the prevalence of *Sarcocystis* sporocysts in wild canids. *Sarcocystis* sporocysts were detected in feces of 21 of 150 (8.0%) coyote feces from Utah and Idaho (Fayer and Johnson, 1975) and in feces of 89 of 169 (52.7%) coyotes from Montana (Dubey *et al*., 1978). In both studies, sporocysts were infectious to cattle.

### Molecular characterization

Based on our molecular results, we found 100% identity in all the 4 genes from experimentally infected calf no. 383 with other *S. cruzi* spp., confirming that the life stages described belong to *S. cruzi*.

## Supporting information

Dubey et al. supplementary material 1 Dubey et al. supplementary material

Dubey et al. supplementary material 2 Dubey et al. supplementary material

Dubey et al. supplementary material 3 Dubey et al. supplementary material

## Data Availability

The slide specimens were deposited in United States National Parasite Collection in the Division of Invertebrate Zoology and National Museum of Natural History, Smithsonian Institution, Museum Support Center, MRC 534, 4210 Silver Hill Road, Suitland, Maryland 20746, USA, under numbers USNM 1606783-1606795. The molecular sequences obtained in this study were deposited to GenBank under accession numbers OR378481, OR378483, OR428752 and OR428753 for *Sarcocystis cruzi*.

## Supplementary material

The supplementary material for this article can be found at https://doi.org/10.1017/S003118202300094X
