# Supplementary material for: Sarcocystis cruzi (Hasselmann, 1923) Wenyon, 1926: redescription, molecular characterization and deposition of life cycle stages specimens in the Smithsonian Museum
Source: Parasitology. 2023 Oct 18;150(13):1192–206. doi: 10.1017/S003118202300094X (PMC10941222; doi:10.1017/S003118202300094X)
Supplement: Dubey et al. supplementary material 1 — Dubey et al. supplementary material [file S003118202300094Xsup001.docx]

Table S1. Summary of experiments with *Sarcocystis cruzi* infections in cattle performed at Beltsville.

| No. of sporocysts | No. of calves | Outcome of infection | Stage of *Sarcocystis* | References |
| --- | --- | --- | --- | --- |
| 250,000-1,000,000 | 6 | Acute sarcocystosis, died or euthanized day 26-33 | Schizonts | Fayer and Johnson (1973) |
| 220,000-1,000,000 | 20 | Acute sarcocystosis, died or euthanized day 26-54 | Schizonts, immature sarcocysts | Fayer and Johnson (1974)**;** Johnson *et al.* (1975) |
| 200,000 | 8 | Acute sarcocystosis, chemotherapy with amprolium effective | Schizonts, immature sarcocysts | Fayer and Johnson (1975); Johnson *et al.* (1975) |
| 300,000-1,000,000 | 6 pregnant cows | Abortion, fetal mortality, acute sarcocystosis | Sarcocysts | Fayer *et al.* (1976) ; Stalheim *et al.* (1976) |
| 200,000 | 8 | Blood chemistry and serology studied | Acute | Fayer and Lunde (1977) |
| 200,000 | 3 | Pyrexia | First generation schizonts | Fayer (1977) |
| 2, 500, 000 | 1 | Acute phase | Ultrastructure of second generation schizonts | Pacheco and Fayer (1977) |
| 150,000-1,000,000 | Not stated | Acute phase | Ultrastructure of  Immature sarcocysts | Pacheco *et al.* (1978) |
| 350,000 | 1 | Vascular phase | Merozoites in blood | Fayer (1979) |
| 380,000 | 1 | Vascular phase | Transmission by blood transfusion | Fayer and Leek (1979) |
| 200,000 | 5 | Growth factors | Not studied | Fayer and Lynch (1979) |
| 200,000 | 4 | Acute sarcocystosis, pathogenesis of anemia | Not studied | Fayer and Prasse (1981) |
| 60,000 or 120,000 | 8 pregnant cows | Acute sarcocystosis, no parasites in colostrum | Not studied | Fayer *et al.* (1982) |
| 60,000 or 120,000 | 8 pregnant cows | Acute sarcocystosis, lower milk yield | Not studied | Fayer *et al.* (1983) |
| 50,000-100,000 | 24 | Mild symptoms | Not studied | Fayer and Dubey (1984) |

**Fayer R** (1977). The first asexual generation in the life cycle of *Sarcocystis bovicanis*. *Proceedings of the Helminthological Society of Washington*. 44, 206-209.

**Fayer R** (1979). Multiplication of *Sarcocystis bovicanis* in the bovine bloodstream. *Journal of Parasitology*. 65, 980-982.

**Fayer R and Dubey JP** (1984). Protective immunity against clinical sarcocystosis in cattle. *Veterinary Parasitology*. 15, 187-201.

**Fayer R and Johnson AJ** (1973). Development of *Sarcocystis* *fusiformis* in calves infected with sporocysts from dogs. *Journal of Parasitology*. 59, 1135-1137.

**Fayer R and Johnson AJ** (1974). *Sarcocystis* *fusiformis*: Development of cysts in calves infected with sporocysts from dogs. *Proceedings of the Helminthological Society of Washington*. 41, 105-108.

**Fayer R and Johnson AJ** (1975). Effect of amprolium on acute sarcocystosis in experimentally infected calves. *Journal of Parasitology*. 61, 932-936.

**Fayer R and Leek RG** (1979). *Sarcocystis* transmitted by blood transfusion. *Journal of Parasitology*. 65, 890-893.

**Fayer R and Lunde MN** (1977). Changes in serum and plasma proteins and in IgG and IgM antibodies in calves experimentally infected with *Sarcocystis* from dogs. *Journal of Parasitology*. 63, 438-442.

**Fayer R and Lynch GP** (1979). Pathophysiological changes in urine and blood from calves experimentally infected with *Sarcocystis cruzi*. *Parasitology*. 79, 325-336.

**Fayer R and Prasse KW** (1981). Hematology of experimental acute *Sarcocystis bovicanis* infection in calves. I. Cellular and serologic changes. *Veterinary Pathology*. 18, 351-357.

**Fayer R, Johnson AJ, and Lunde M** (1976). Abortion and other signs of disease in cows experimentally infected with *Sarcocystis* *fusiformis* from dogs. *Journal of Infectious Diseases*. 134, 624-628.

**Fayer R, Leek RG, and Lynch GP** (1982). Attempted transmission of *Sarcocystis bovicanis* from cows to calves via colostrum. *Journal of Parasitology*. 68, 1127-1129.

**Fayer R, Lynch GP, Leek RG, and Gasbarre LC** (1983). Effects of sarcocystosis on milk production of dairy cows. *Journal of Dairy Science*. 66, 904-908.

**Johnson AJ, Hildebrandt PK, and Fayer R** (1975). Experimentally induced *Sarcocystis* infection in calves: pathology. *American Journal of Veterinary Research*. 36, 995-999.

**Pacheco ND and Fayer R** (1977). Fine structure of *Sarcocystis cruzi* schizonts. *Journal of Protozoology*. 24, 382-388.

**Pacheco ND, Sheffield HG, and Fayer R** (1978). Fine structure of immature cysts of *Sarcocystis cruzi*.  *Journal of Parasitology*. 64, 320-325.

**Stalheim OH, Proctor SJ, Fayer R, and Lunde M** (1976). Death and abortion in cows experimentally infected with *Sarcocystis* from dogs. *19th Annual Proceedings of the American Association of Veterinary Laboratory Diagnosticians*. 317-327.
