# Supplementary material for: Sarcocystis cruzi (Hasselmann, 1923) Wenyon, 1926: redescription, molecular characterization and deposition of life cycle stages specimens in the Smithsonian Museum
Source: Parasitology. 2023 Oct 18;150(13):1192–206. doi: 10.1017/S003118202300094X (PMC10941222; doi:10.1017/S003118202300094X)
Supplement: Dubey et al. supplementary material 2 — Dubey et al. supplementary material [file S003118202300094Xsup002.docx]

TABLE S2. Developmental stages in calves fed sporocysts of *Sarcocystis cruzi* from coyotes.

| **Calf No.** | **Experiment No.** | **Histology Case No.** | **Dose (x1000)** | ***S. cruzi* Isolate (see Table S3)** | **Necropsy (day post-inoculation)** | **Blood Parasitemia** | | **Tissue stages** | |
| --- | --- | --- | --- | --- | --- | --- | --- | --- | --- |
|  |  |  |  |  |  | **Day(s) examined** | **Day(s) positive** | **Stage** | **Tissues** |
| 522 | 64 | 271‐81 | 500,000 | 1a | 4 K | 1‐4 | None | Sporozoite | ML |
| 55 | 3 | 19254 | 50,000 | 7 | 4 K | 4 | None | None |  |
| 66 | 6 | 19281 | 50,000 | 6 | 7 K | 7 | None | None |  |
| 357 | 28 | 354‐80 | 500,000 | 1‐3 | 7 K | ND |  | Sporozoite, I |  |
| 36 | 3 | 19252 | 50,000 | 7 | 11 K | ND |  | I | ML |
| 38 | 3 | 19253 | 50,000 | 7 | 15 K | ND |  | I | Several |
| 226 | 31 | 399‐80 | 50,000 | 4 | 15 K | ND |  | I | Ki |
| 593 | 72 | 421‐81 | 150,000 | 1a | 15 K | 15 | None | I | Several |
| 380 | 35 | 412‐80 | 50,000 | 5 | 17 K | 17 |  | I | Several |
| 379 | 35 | 415‐80 | 50,000 | 5 | 19 K | 19 | None | I, II | MK, Ki |
| 54 | 5 | 19257 | 50,000 | 7 | 22 K | ND |  | I, II | Several |
| 383 | 35 | 451‐80 | 150,000 | 5 | 24 K | 6‐24 | 24 | I, II | Several |
| 359 | 28 | 357‐80 | 50,000 | 1‐3 | 25 K | 12‐25 | None | I, II | Several |
| 598 | 72 | 417‐81 | 150,000 | 1a | 26 K | 15‐26 | 26 | I, II | Several |
| 68 | 6 | 19296 | 50,000 | 6 | 28 KM | 28 | 28 | II, Mz | Several |
| 358 | 28 | 358‐80 | 25,000 | 1‐3 | 29 KM | 12‐29 | 27‐29 | II, Mz | Several |
| 70 | 6 | 19298 | 50,000 | 6 | 29 D | ND | ND | II | Several |
| 418 | 47 | 45‐81 | 2500 | 2 | 31 D | 14‐31 | 25‐31 | II | Several |
| 600 | 69 | 415‐81 | 10,000 | 1a | 31 D | 22, 24, 28, 29 | 28, 29 | II | Several |
| 378 | 37 | 452‐80 | 2000 | 3 | 32 K | 30‐32 | 30, 31 | II | Ki, Sk, H |
| 420 | 47 | 46‐81 | 2500 | 2 | 33 K | 14‐33 | 26, 28, 31‐33 | II | ES, H |
| 412 | 45 | 54‐81 | 2000 | 3 | 34 D | 14‐34 | 25‐34 | II, Mz | H, Sk, Ki, T |
| 419 | 47 | 128‐81 | 2500 | 2 | 35 D | 14‐35 | 25, 27, 29‐35 | II, Mz | H, Di, T, Ki |
| 356 | 28 | 360‐80 | 1000 | 1‐3 | 35 KM | 12‐35 | 28, 30 | Mz | H, T |
| 381 | 33 | 455‐80 | 5000 | 3 | 35 KM | ND |  | II, Mz | T, H, Sk, Ru |
| 478 | 53 | 170‐81 | 275 | 2 | 38 KM | ND |  | Mz | H |
| 40 | 3 | 19256 | 100 | 7 | 41 K | ND |  | Mz | H, Di |
| 601 | 69 | 469‐81 | 1000 | 1a | 42 D | 22, 24, 28‐41 | 28‐41 | Mz, II | H |
| 461 | 52 | 161‐81 | 774 | 3 | 43 D | 26‐42 | 26‐42 | II, Mz | H |
| 388 | 40 | 26‐81 | 1000 | 3 | 44 K | 16‐44 | 26, 28‐32, 34,  38 | Mz | H |
| 479 | 53 | 174‐81 | 55 | 2 | 45 KM | ND |  | Sarcocysts,  Mz | H, Sk |
| 421 | 48 | 155‐81 | 287 | 1 | 46 K | 28‐30, 46 | 46 | II, Sarcocysts | H, Sk, T |
| 460 | 52 | 171‐81 | 287 | 3 | 55 K | 26‐47, 55 | 26‐46 | Sarcocysts | H, T, Ru, O, Ub,  In, Ao |
| 39 | 3 | 19282 | 100 | 7 | 67 K | ND |  | Sarcocysts | Muscles |
| 422 | 48 | 176‐81 | 287 | 1 | 86 K | 28‐30 | None | Sarcocysts | Muscles |
| 71 | 6 | 13‐80 | 1000 | 7 | 90 D | ND |  | Sarcocysts | Muscles, Br, Sp |
| 489 | 57 | 399‐81 | 287 | 1 | 112 K | ND |  | Sarcocysts | Muscles |
| 488 | 56 | 414‐81 | 100 | 2 | 153 K | ND |  | Sarcocysts | Muscles |

Ao=abomasum, Br=brain, D=died, Di=diaphragm, E=euthanized, Em=euthanized when moribund, Es=esophagus, H=heart, In=intestine, Ki=kidney, ML=mesenteric lymph nodes, Mz=Merozoites, ND=no data, O=omasum, Ru=rumen, Sk=skeletal muscle from thigh, Sp=spinal cord, T=tongue, Ub=urinary bladder, I=first generation meronts, II=second generation meronts.
