# Supplementary material for: Sarcocystis cruzi (Hasselmann, 1923) Wenyon, 1926: redescription, molecular characterization and deposition of life cycle stages specimens in the Smithsonian Museum
Source: Parasitology. 2023 Oct 18;150(13):1192–206. doi: 10.1017/S003118202300094X (PMC10941222; doi:10.1017/S003118202300094X)
Supplement: Dubey et al. supplementary material 3 — Dubey et al. supplementary material [file S003118202300094Xsup003.docx]

Table S3. *Sarcocystis cruzi* infection in laboratory raised coyotes fed naturally infected beef.

| Coyote  No. | Infected Beef  Fed | Date Fed (MM/DD/YYYY) | Euthanized Day p.i. | Prepatent Period (Day) | Sporocysts  In Feces | Sporocysts in Digest | *S. cruzi*  isolate No. |
| --- | --- | --- | --- | --- | --- | --- | --- |
| 5 | Natural^a^ | 08/02/1979 | 13 | 9 | 6,000.000 | 195,000,000 | B7 |
| 7 | Natural^a^ | 05/25/1979 | 27 | 9 | 17,875,000 | 7,500,000 |  |
| 8 | Natural^a^ | 05/25/1979 | 17 | 9 | 10,625,000 | 4,500,000 |  |
| 16 | Natural^a^ | 09/27/1979 | 13 | 9 | 500,000 | 112,500,000 | B6 |
| 21 | Natural^a^ | 06/26/1980 | 12 | 9 | No data | >10,000,000 | B2 |
| 23 | Natural^a^ | 07/03/1980 | 12 | 9 | No data | >100,000,000 | B3 |
| 25 | Natural^a^ | 07/11/1980 | 14 | 9 | No data | >100,000,000 | B1 |
| 26 | Natural^a^ | 09/12/1980 | 12 | 9 | No data | 50,000,000 | B4 |
| 27 | Natural^a^ | 10/02/1980 | 13 | 9 | No data | 265,000,000 | B5 |
| 30 | Experimental^b^ | 07/11/1980 | 12 | 9 | No data |  |  |
| 46 | Experimental^c^ | 09/11/1981 |  | 9 | No data | 300,000,000 | 1a |
| 43 | Experimental^c^ | 06/03/1981 | 16 | 9 | No data | >100,000.000 |  |
| 38 | Experimental^c^ | 06/03/1981 | 16 | 9 | No data | >100,000,000 |  |
| 33 | Experimental^c^ | 06/03/1981 | 16 | 9 | No data | No data |  |
| 34 | Experimental^c^ | 06/03/1981 | 16 | 9 | No data | No data |  |
| 44 | Experimental^c^ | 06/03/1981 | 16 | 9 | No data | No data |  |

^a^ From different cows in Bozeman, Montana.

^b^ From calf inoculated with sporocysts of the Beltsville isolate of *S. cruzi*.

^c^ From calves inoculated with sporocysts from coyote 25 of the Bozeman isolate of *S. cruzi*.
